# Optimizing a human forensic STR panel for non-invasive genetic monitoring of chimpanzees (*Pan troglodytes*)

**DOI:** 10.1186/s13104-026-07997-y

**Published:** 2026-09-09

**Authors:** Shrutarshi Paul, Fabrice Kentatchime, Tyler Andres-Bray, Tista Ghosh, Giovana Naira Pereira Couto, Kevin Njabo, Mary Katherine Gonder

**Affiliations:** 1 Department of Ecology and Conservation Biology, Texas A&M University, College Station, TX USA; 2 Department of Ecology and Evolutionary Biology, University of California, Los Angeles, CA USA; 3 Department of Ecosystem Science and Management, University of Northern British Columbia, Prince George, British Columbia, Canada

**Keywords:** Chimpanzee genetics, Microsatellites, STRs, Non-invasive sampling, Fecal DNA, GlobalFiler™, Genotyping error, Conservation genetics, Cameroon, wildlife forensics

## Abstract

**Objective:**

Accurate molecular genotyping is essential for conservation genetics, population monitoring, pedigree reconstruction, and wildlife forensic investigations in endangered primates. Although SNP-based approaches are increasingly available, microsatellites remain among the most practical tools for non-invasive individual identification because of their high allelic diversity and strong discriminatory power from relatively few loci. Here, we present a standardized optimization framework for adapting the human forensic GlobalFiler™ PCR Amplification Kit to non-invasive chimpanzee genotyping.

**Results:**

Using blood and fecal DNA from captive and wild chimpanzees in Cameroon, we integrated DNA quality screening, chimpanzee-specific allele binning, replicate consensus genotyping, statistical evaluation of locus performance, and composite marker ranking to develop a robust 16-locus panel for individual identification. Estimated marginal means were calculated from the fitted beta regression models to assess amplification success, allelic dropout, and false allele rates across sample types and loci. These metrics, combined with measures of discriminatory power, identified D10S1248, FGA, D2S441, and D3S1358 as the highest-performing loci. The optimized panel achieved 95.4% amplification success with low allelic dropout (0.08) and false allele (0.02) rates. It identified 64 unique individuals (39 males and 25 females) and showed high discriminatory power (P_ID(sibs)_ = 1.2 × 10⁻⁶). This workflow is easily transferable and can complement SNP genotyping, metabarcoding, microbiome analyses, and other molecular approaches in a wide range of non-invasive genetic studies.

**Supplementary Information:**

The online version contains supplementary material available at https://doi.org/10.1186/s13104-026-07997-y.

## Introduction

Reliable genetic identification is fundamental to conservation genetics, enabling individual identification, pedigree reconstruction, forensic investigations, and assessments of genetic diversity and population structure [1, 2]. This is particularly important in Cameroon, where genetic monitoring combines samples from wild populations and rescued captive chimpanzees of uncertain ancestry, creating additional challenges for individual identification and population-level inference [3]. Chimpanzee monitoring increasingly relies on non-invasive fecal sampling, which avoids animal capture but often yields degraded, inhibitor-rich DNA that causes amplification failure and genotyping errors [4, 5].

Although single nucleotide polymorphism (SNP) based approaches are becoming more common, microsatellite markers remain widely used for individual identification because their high allelic diversity provides strong discriminatory power from relatively few loci and perform well with degraded DNA [6, 7]. Several commercial human forensic short tandem repeat (STR) systems have demonstrated successful cross-amplification in chimpanzees and other great apes, but mostly with tissue samples [8–10]. However, their performance on non-invasive fecal samples remains largely untested, emphasizing the need for standardized optimization and locus-level validation using degraded and high-quality chimpanzee DNA [11]. We therefore evaluated and optimized the commercially available human GlobalFiler™ STR system for non-invasive chimpanzee genetic monitoring using blood and fecal samples from captive and wild populations in Cameroon. This STR system has been tested by Singh et al. [9] in chimpanzees using only high-quality tissue samples. We further extended this work by developing a standardized optimization workflow that integrates DNA quality screening, chimpanzee-specific allele binning, replicate consensus genotyping, statistical evaluation of genotyping performance, and locus-ranking based on both technical reliability and discriminatory power. This framework provides a reproducible approach for adapting commercial forensic STR systems to non-invasive wildlife genetics studies and is readily transferable to other applications in great apes and wildlife conservation genetics.

## Methods

**Fig. 1:**
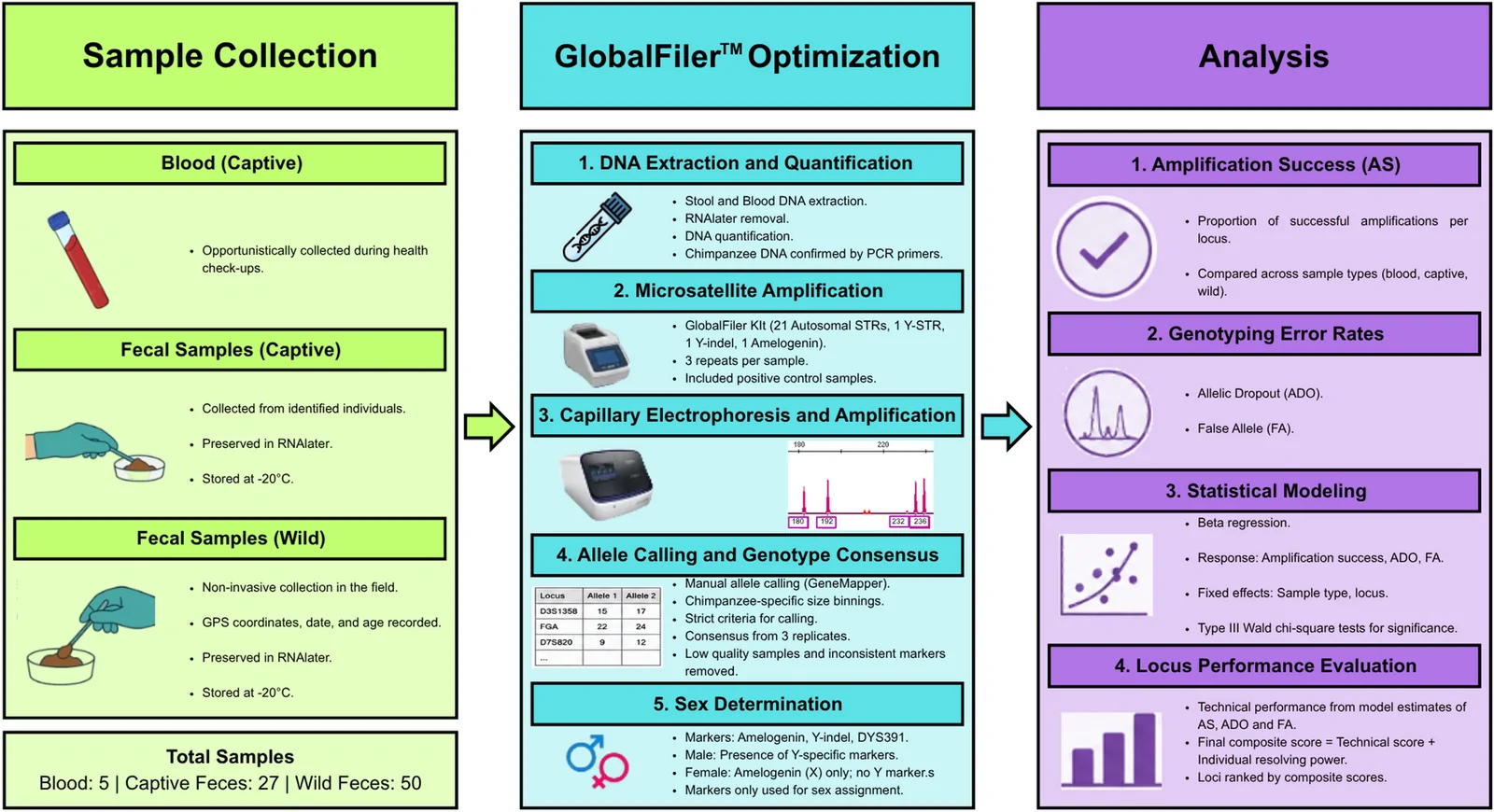
Overview of the methodological pipeline used to optimize the human GlobalFiler™ STR system for chimpanzee non-invasive genetic samples

The entire methodological framework for Globalfiler™ kit optimization is summarized in Fig. 1.

### Sample collection

The study included five blood samples and 77 fecal samples from captive and wild chimpanzees in Cameroon. Blood and 27 fecal samples were collected from captive individuals at the Limbe Wildlife Centre during routine veterinary examinations or shortly after defecation, while 50 fecal samples were collected non-invasively from wild chimpanzees in the Ganga Research Station landscape within Mbam and Djerem National Park. Fecal samples were preserved in RNAlater^®^, transported to the United States under appropriate Convention on International Trade in Endangered Species of Wild Fauna and Flora (CITES) and governmental permits, and stored at − 20 °C until laboratory processing.

### DNA extraction and quantification

Genomic DNA was extracted from blood samples using the Gentra Puregene Blood Kit (Qiagen) and from fecal samples using the QIAamp DNA Stool Mini Kit (Qiagen) following established protocols for great ape samples [3, 12–15]. DNA concentration and purity were assessed using a NanoDrop spectrophotometer and Qubit fluorometer. The presence of endogenous chimpanzee DNA in fecal extracts was confirmed by polymerase chain reaction (PCR) amplification of the mitochondrial HVR1 region [15], followed by chimp DNA quantification using the Quantifiler™ HP and Trio DNA Quantification Kit (Applied Biosystems) [13].

### Globalfiler™ PCR amplification and genotyping

Samples with acceptable DNA purity and containing ≥ 0.05 ng/µL endogenous chimpanzee DNA (manufacturer’s recommended input threshold for the GlobalFiler™ kit) were genotyped using the GlobalFiler™ PCR Amplification Kit (Applied Biosystems), which amplifies 24 genetic markers (21 autosomal STRs, one Y-STR, one Y-indel, and Amelogenin). PCRs were performed according to the manufacturer’s protocol using 500 pg of template DNA per 25 µL reaction, with all samples amplified in triplicate alongside appropriate positive controls. Amplified products were mixed with GeneScan™ 600 LIZ™ Size Standard v2.0 and HidiFormamide following Globalfiler guidelines and separated on a SeqStudio™ Genetic Analyzer (Applied Biosystems).

### Allele calling and genotype consensus

All 24 GlobalFiler™ markers were initially evaluated using five blood samples and 27 captive fecal samples before applying the optimized marker set to 50 wild fecal samples. Fragment analysis was performed in GeneMapper™ v6 (Applied Biosystems), and one blood sample genotype was used as an internal reference to standardize allele calling across runs.

Because GlobalFiler™ was developed for human STR loci, its allele nomenclature cannot be directly applied to chimpanzees. Therefore, alleles were defined using observed fragment lengths (bp), and locus-specific allele bins were manually established from reproducible fragment-size distributions while accommodating microvariants (Additional file 4). No modifications were made to allele bins for the three sex-linked markers.

Alleles with relative fluorescence unit (RFU) values < 75 or exhibiting multiple, inconsistent, or missing peaks were excluded from further analyses [16, 17]. Heterozygous genotypes were accepted only when the smaller peak was at least 50% of the height of the larger peak [18, 19]. All samples were independently amplified three times, and consensus genotypes were generated using the Quality Index approach, requiring agreement between at least two of three replicates [20, 21].

### Data analysis 

#### Amplification success, genotyping error rates, individual and sex identification

Out of 24 markers, only autosomal markers were evaluated for amplification success and genotyping error rates. Three sex-linked markers (Amelogenin, Y-indel, and DYS391) were used exclusively for sex determination and were not included in the analyses of genotyping error rates or locus performance [22]. Samples producing consensus genotypes at fewer than 80% of loci were excluded from downstream locus-performance analyses to ensure that comparisons among loci reflected marker performance rather than variation caused by extremely poor sample quality. Amplification success (AS) and genotyping error rates such as False allele (FA) and Allelic dropouts (ADO) were calculated manually for the final selected autosomal panel by following previously published recommendations [23–25] for each locus using replicate data. GenAlEx 6.5 [26] was used to calculate basic genetic diversity indices, while Cervus v3.0.7 [27] was used to estimate the polymorphic information content (PIC) and probability of identity among siblings (P_ID(sibs)_) to assess individual identification power.

#### Modeling variation in genotyping performance across sample types and loci

To test whether the observed variation in genotyping performance was associated with sample type and locus, statistical analyses were conducted using beta regression models with a logit link function implemented in the glmmTMB package in R [28]. Separate models were fitted for AS, ADO, and FA, with sample type and locus included as fixed effects. Because the response variables were bounded proportions (0–1), values equal to 0 or 1 were slightly adjusted prior to analysis to satisfy the assumptions of the beta distribution [29]. Type III Wald chi-square tests were used to evaluate the significance of model terms. Estimated marginal means (EMMs) for AS, ADO and FA were obtained using the emmeans package in R for sample type and locus [30, 31].

#### Locus performance scoring and ranking

Our ranking approach follows the general concept of composite-index construction, where multiple indicators are normalized to a common scale and then combined to facilitate comparative ranking [32]. Estimated marginal means (EMMs) for AS, ADO, and FA rates were extracted from the beta regression models for each locus and min-max normalized. A technical performance score was then calculated by combining normalized EMMs, with amplification success contributing positively and allelic dropout and false allele rates contributing equally as penalties (Technical Score = EMM_(AS)_-(EMM_(ADO)_ + EMM_(FA)_)). The P_ID(sibs)_ was subsequently min–max normalized to ensure comparable weighting with the technical score and used as a penalization to the technical performance score to generate a final composite score (Final score=Technical Score-(P_ID(sibs)_)) reflecting both technical reliability and discriminatory power allowing loci to be ranked accordingly.

## Results

### Selection of final set of STR loci

Three poorly amplified reference samples (< 30% of loci amplified) were excluded prior to marker evaluation. Based on amplification performance and peak characteristics, five loci were removed: SE33 and D18S51 failed to amplify, D1S1656 exhibited consistent non-specific amplification, and D19S433 and D12S391 showed low amplification success (< 50%). The remaining 16 loci were validated using 50 field-collected fecal samples, and final allele bins were optimized for chimpanzees.

### Summary statistics, marker performance, individual and sex identification

Consistent with the predefined filtering criteria described above, samples amplifying at fewer than 80% of loci (< 13 of 16 loci) were excluded, resulting in a dataset of 69 samples (40 field-collected fecal, 24 sanctuary-collected fecal, and five blood samples). Overall, the 16-locus panel showed high genotyping performance, with a mean AS of 95.4% and low mean ADO (ADO = 0.08) and FA (FA = 0.02) rates. Genotyping performance varied by sample type, with field-collected fecal samples showing the highest error rates, followed by sanctuary-collected fecal samples, while blood samples exhibited negligible error. The panel showed moderate genetic diversity (mean observed heterozygosity = 0.55; mean number of alleles = 8 and mean PIC = 0.67), with six loci deviated from Hardy–Weinberg equilibrium (HWE) (Table 1).

**Table 1:** Locus-wise summary statistics of the chimpanzee STR panel used for genotyping chimpanzee samples

Locus	Number of Alleles	AS (%)	ADO (95% CI)	FA (95% CI)	Ho	He	PID_SIBS_	PIC	Final Composite Score
D10S1248	9	100	0.02 (0.06 − 0.004)	0 (0.02-0)	0.7	0.75	0.398	0.71	0.903
FGA	11	100	0.04 (0.09 − 0.02)	0.01 (0.03 − 0.001)	0.71	0.79	0.368	0.77	0.707
D2S441	8	99.5	0.05 (0.1 − 0.02)	0.01 (0.03 − 0.001)	0.74	0.84	0.342	0.82	0.689
D3S1358	8	98.6	0.025 (0.06 − 0.01)	0.01 (0.04 − 0.003)	0.78	0.75	0.401	0.71	0.680
D13S317	9	99.5	0.05 (0.11 − 0.03)	0.01 (0.03 − 0.001)	0.71	0.78	0.380	0.74	0.646
D21S11*	11	97.6	0.04 (0.09 − 0.01)	0.01 (0.04 − 0.003)	0.65	0.83	0.343	0.81	0.591
D8S1179*	11	96.6	0.05 (0.11 − 0.02)	0.02 (0.05 − 0.01)	0.58	0.84	0.339	0.82	0.491
VWA*	10	97.1	0.06 (0.13 − 0.03)	0.025 (0.06 − 0.01)	0.54	0.77	0.381	0.74	0.397
D5S818	4	96.6	0.02 (0.08 − 0.003)	0.01 (0.03-0001)	0.46	0.56	0.540	0.46	0.388
D22S1045*	6	87	0.06 (0.15 − 0.02)	0 (0.02-0)	0.43	0.72	0.417	0.69	0.305
D2S1338	11	90.3	0.05 (0.11 − 0.02)	0.06 (0.10 − 0.03)	0.67	0.8	0.361	0.78	0.200
D16S539	7	96.1	0.12 (0.23 − 0.09)	0.025 (0.06 − 0.01)	0.6	0.73	0.409	0.69	0.189
CSF1PO*	10	94.2	0.13 (0.23 − 0.08)	0.08 (0.12 − 0.04)	0.59	0.83	0.348	0.8	0.020
TPOX*	7	92.8	0.25 (0.54 − 0.21)	0.06 (0.10 − 0.03)	0.25	0.59	0.503	0.55	-0.265
D7S820	5	82.1	0.06 (0.17 − 0.01)	0.03 (0.07 − 0.01)	0.32	0.42	0.627	0.38	-0.39
TH01	3	98.6	0.3 (0.88 − 0.12)	0.01 (0.03-0)	0.04	0.04	0.956	0.04	-0.581

The table summarizes the number of observed alleles, amplification success rate (AS), allelic dropout (ADO), false allele (FA) rate, observed heterozygosity (Ho), expected heterozygosity (He), probability of identity among siblings (P_ID(sibs)_), polymorphic information content (PIC) and the final composite score for each locus. Loci are ranked according to composite performance, integrating amplification success, genotyping error rates, and discriminatory power, with higher scores indicating greater overall suitability. Confidence intervals (CI) are calculated based on Clopper–Pearson Exact test. Loci showing deviation from HWE are highlighted by *

Unique individuals were identified by requiring matches at ≥ 13 loci while allowing up to two mismatches to account for genotyping errors. This identified two exact-match pairs and six fuzzy-match pairs, corresponding to three recaptured individuals (39R1CMMD, three recaptures; 30R1CMMD, one recapture; and CMSY01R1, one recapture; Additional file 3). Across the final dataset, 64 unique individuals were identified with low cumulative probabilities of identity (P_ID(sibs)_ = 1.2 × 10⁻⁶). Molecular sexing was successful for all 69 samples (43 males and 26 females), and detailed results for marker amplification, concordance, and sex assignments are presented in Table 2.

**Table 2:** Summary of molecular sexing performance and validation results for 69 chimpanzee samples

Category	Parameter	Value
**Marker performance**	Y indel amplification success	69/69 (100.0%)
	Amelogenin amplification success	67/69 (97.1%)
	DYS391 amplification success	57/69 (82.9%)
**Sex assignment results**	Total samples	69
	Males	43
	Females	26
	Concordance across three markers	60
	Concordance between two markers only	9
**Validation**	Concordance with recorded sex	97%

The table presents amplification success of the three sex-linked markers (Y indel, Amelogenin, and DYS391), sex assignment outcomes, marker concordance, and agreement with observer-recorded sex

### Model-based evaluation of genotyping performance across sample types and loci ranking

Genotyping performance differed significantly among sample types and microsatellite loci across all three metrics (AS, ADO, and FA rates; Type III Wald χ² tests, *P* < 0.05; Additional file 1). Sample type had a strong effect on amplification success, with blood samples showing the highest success rate, followed by sanctuary-derived and field-collected fecal samples. Similarly, ADO and FA rates were lowest in blood and highest in field-collected fecal samples. Locus identity also significantly influenced genotyping performance, though the magnitude of the effects varied across metrics. Estimated marginal means revealed substantial heterogeneity among loci in AS, ADO and FA (Additional file 2). Several loci, including D10S1248, D3S1358, FGA, and D5S818, consistently performed well, whereas D7S820, CSF1PO and TPOX performed poorly across metrics.

Overall, marker ranking based on combined genotyping and power of resolution revealed a clear hierarchy of loci with D10S1248, D22S441, FGA, and D3S1358 among the highest-performing loci, and TH01, TPOX, CSF1PO, and D7S820 among the lowest. Intermediate performance was observed for the remaining loci (Table 1).

## Discussion and conclusion

This study identified a stable 16-locus panel from the 21 autosomal loci included in the GlobalFiler™ PCR Amplification Kit for non-invasive chimpanzee genotyping. Most loci showed consistent amplification and genotyping performance across sample types, whereas D18S51, D19S433, and D1S1656 consistently performed poorly and were excluded. This pattern is broadly consistent with previous evaluations of human forensic STR systems in chimpanzees, although the specific loci showing poor amplification have varied among studies [9, 33–35]. Such variation is expected when human-derived primers are applied across species and when DNA quantity and quality differ among samples. A previous evaluation of GlobalFiler in chimpanzee tissue samples reported successful amplification of 18 of the 21 autosomal loci, highlighting the utility of the kit in identifying individual chimpanzees [9]. Our results extend these observations by evaluating performance across a larger and more heterogeneous set of non-invasive fecal samples.

The heterogeneity in the dataset enabled more robust locus assessment than earlier studies, reducing bias from small sample sizes and stochastic amplification failure. For example, one study [9] classified D5S818 as a failed locus, likely due to stochastic non-amplification associated with a very small sample size (*n* = 3). In contrast, loci in the present study were not designated as failed solely based on raw amplification patterns; instead, poorly amplified samples (< 80% AS) were excluded prior to locus-level assessment to reduce false-negative inflation. The exclusion of severely degraded samples was intended to evaluate locus performance under realistic analytical conditions rather than allow a small number of extremely poor-quality samples to disproportionately influence estimates of marker reliability. The observed and expected heterozygosity were broadly comparable across loci, indicating substantial moderate variation within the sampled individuals, comparable with other studies [8, 9, 12]. Departures from HWE for six loci should not necessarily be interpreted as evidence of biological processes such as selection or inbreeding as our dataset combines samples from captive and wild chimpanzees and was primarily assembled for marker validation rather than population genetic inference. The very low cumulative PID(sibs) of 1.2 × 10⁻⁶ indicates strong discriminatory power for individual identification and is consistent with the levels reported in previous non-invasive genetic studies of chimpanzees [1].

We also developed chimpanzee-specific fragment-size bins to improve consistency in allele assignment across PCR replicates, runs, and samples of varying DNA quality. Studies have highlighted the challenges of using human STR kits in non-human primates and the need for species-specific calibration [9, 34, 35]. In the present study, we provide empirically derived allele size ranges for the GlobalFiler™ panel in chimpanzees, based on both captive and wild fecal samples. These bins facilitate consistent allele assignment and provide a reference for allele size ranges and their distribution that may be useful in future chimpanzee genetic studies.

Beyond marker selection, we implemented a locus-ranking framework combining amplification success, genotyping error rates (ADO, FA), and discriminatory power (P_ID(sibs)_). This model-based approach accounts for variation in sampling effort, sample quality and replication effort, providing a more robust assessment than raw error counts alone. Incorporating locus-specific discriminatory power (P_ID(sibs)_) further strengthened the ranking by integrating technical reliability with individual identification capacity. For example, TH01 exhibited one of the highest allelic dropout rates and relatively poor discriminatory power, which resulted in a low overall ranking despite exhibiting high amplification success and low false allele rates. Studies of wild chimpanzees have similarly shown that allelic dropout can be substantial in low-template samples and that repeated PCRs are required to obtain reliable consensus genotypes [1, 36]. Conversely, D10S1248 ranked first despite having lower individual resolving power than many loci because of its consistently superior performance across the technical metrics. This shows that a locus with moderate discriminatory power can be highly useful when it is reliably amplified across degraded samples. The examples provided above demonstrate that locus suitability cannot be adequately assessed using any single metric alone, specifically when working with non-invasive samples.

The optimization approach in this study integrates DNA quality assessment, species-specific allele calibration, replicate consensus genotyping, evaluation of genotyping performance, and locus ranking, providing a framework that may be useful for evaluating commercial forensic STR panels for non-invasive genetic monitoring. Its applicability to other species, marker systems, and sampling contexts would require separate validation and has potential to complement SNP genotyping, metabarcoding, and other genomic approaches. Taken together, the 16-locus panel balances amplification reliability, genotyping accuracy, and discriminatory power, providing a practical basis for individual chimpanzee identification and genetic monitoring from non-invasive samples while retaining the flexibility of a commercially available forensic STR system.

## Limitations

Validation was restricted to chimpanzees from Cameroon, which may have resulted in population-specific allele frequencies, deviations from HWE, low polymorphisms in some loci and underrepresentation of genetic variation. Because the dataset was not designed to represent the full geographic and genetic diversity of chimpanzees, these findings should be interpreted cautiously, and the performance and allele ranges of these loci should be further evaluated using samples from additional populations and geographic regions in future studies. Although a standard 25 µL reaction volume was used initially, successful amplification was also achieved with 18 and 12.5 µL reactions, suggesting further optimization of reaction volumes and reagent concentrations according to DNA quality could improve efficiency and reduce costs [37]. In addition, alternative STR kits may show different locus performance depending on marker composition and species compatibility and could provide complementary or improved performance in some contexts. Although consistency was observed across replicate PCRs, different thermocyclers, and runs on different days, formal inter-operator and inter-laboratory validation was not conducted and should be addressed in future studies, specifically for forensic standardizations. The allele bins derived from the chimpanzee samples examined in this study require validation across additional populations and geographic regions and can further be modified accordingly. Finally, although GlobalFiler™ provides a standardized multiplex platform, its cost may limit large-scale ecological applications, highlighting the potential need for smaller chimpanzee-optimized STR panels derived from the locus-ranking framework developed here.

## Supplementary information

Below is the link to the electronic supplementary material.

Supplementary Material 1: Additional file 1: Beta regression estimates for amplification success (AS), allelic dropout (ADO), and false allele (FA) across sample types and microsatellite loci. Values are presented as logit-scale coefficient estimates ± standard error (SE). Bold values indicate statistically significant effects (p < 0.05). Blood samples served as the reference category for sample type comparisons. Locus coefficients are interpreted relative to a model-specific reference locus. The reference loci were D10S1248 for AS, TPOX for ADO, and CSF1PO for FA

Supplementary Material 2: Additional file 2: Estimated marginal means (EMMs) for amplification success, allelic dropout (ADO), and false allele (FA) across microsatellite loci derived from generalized linear mixed models (GLMMs). Values are presented as model-predicted marginal means ± standard error (SE) on the logit scale, averaged across sample types. Higher EMM values for amplification success indicate improved amplification performance, whereas lower EMM values for ADO and FA indicate reduced genotyping error rates

Supplementary Material 3: Additional file 3: Recapture details of exact and fuzzy match pairs

Supplementary Material 4: Comparison of allele binning modifications between a human sample (Panel A) and a chimpanzee sample (Panel B) for three loci. The locus D3S1358 required no modification, whereas VWA and D16S539 required rearrangement of allele bins to accommodate chimpanzee-specific alleles

## Data Availability

Additional data and code supporting the analyses presented in this study are available in Figshare at the following reviewer link: https://figshare.com/s/aa98e130773b397a9b6b. The raw genotyping data are not included because they are part of an ongoing study and will be made publicly available upon completion of that project.

## Declarations

### Artificial intelligence statement

During the preparation of this manuscript, the authors used OpenAI ChatGPT to assist with language editing, manuscript organization, and development of a preliminary graphical abstract concept. All data collection, statistical analyses, interpretation, and final figure preparation were conducted by the authors without the use of AI-generated analyses. The authors reviewed, revised, and approved all content and take full responsibility for the published work.

## Abbreviations

ADO: Allelic dropout
AS: Amplification success
CI: confidence interval
CITES: Convention on International Trade in Endangered Species of Wild Fauna and Flora
EMMs: Estimated marginal means
FA: False allele
HWE: Hardy–Weinberg equilibrium
PCR: Polymerase chain reaction
PIC: polymorphic information content
P_ID(sibs)_: Probability of identity among siblings
RFU: Relative fluorescence unit
SNP: Single nucleotide polymorphism
STR: Short tandem repeat
