# Supplementary material for: Optimizing a human forensic STR panel for non-invasive genetic monitoring of chimpanzees (Pan troglodytes)
Source: BMC Res Notes. 2026 Sep 9;19:388. doi: 10.1186/s13104-026-07997-y (PMC13721693; doi:10.1186/s13104-026-07997-y)
Supplement: Supplementary file 1 [file 13104_2026_7997_MOESM2_ESM.docx]

| **Predictor** | **Amplification success (AS) (β ± SE)** | **Allelic dropout (ADO) (β ± SE)** | **False allele (FA)**  **(β ± SE)** |
| --- | --- | --- | --- |
| Intercept | **5.87 ± 0.37** | **-3.06 ± 0.27** | **-3.65 ± 0.17** |
| Sample Fecal sanctuary | **-0.91 ± 0.18** | 0.64 ± 0.26 | **0.35 ± 0.16** |
| Sample Fecal wild | **-1.97 ± 0.17** | 2.55 ± 0.24 | **1.34 ± 0.14** |
| D10S1248 | Reference | **-2.17 ± 0.45** | **-1.66 ± 0.32** |
| CSF1PO | **-1.49 ± 0.38** | **-0.36 ± 0.30** | Reference |
| D13S317 | -0.33 ± 0.44 | **-1.40 ± 0.37** | **-1.29 ± 0.28** |
| D16S539 | **-1.16 ± 0.39** | **-0.72 ± 0.32** | **-0.69 ± 0.23** |
| D21S11 | **-0.86 ± 0.40** | **-1.56 ± 0.38** | **-1.07 ± 0.26** |
| D22S1045 | **-2.13 ± 0.36** | **-1.39 ± 0.39** | **-1.66 ± 0.32** |
| D2S1338 | **-1.86 ± 0.37** | **-1.49 ± 0.38** | -0.27 ± 0.21 |
| D2S441 | -0.33 ± 0.44 | **-1.43 ± 0.38** | **-1.15 ± 0.27** |
| D3S1358 | -0.48 ± 0.43 | **-2.03 ± 0.43** | **-1.15 ± 0.27** |
| D5S818 | **-1.13 ± 0.39** | **-2.21 ± 0.46** | **-1.29 ± 0.28** |
| D7S820 | **-2.38 ± 0.36** | **-0.82 ± 0.32** | **-0.63 ± 0.23** |
| D8S1179 | **-1.14 ± 0.39** | **-1.43 ± 0.37** | **-0.92 ± 0.25** |
| FGA | 0.00 ± 0.46 | **-1.49 ± 0.38** | **-1.07 ± 0.26** |
| TH01 | -0.63 ± 0.42 | -0.19 ± 0.28 | **-1.29 ± 0.28** |
| TPOX | **-1.66 ± 0.37** | Reference | -0.35 ± 0.21 |
| VWA | **-0.92 ± 0.40** | **-1.17 ± 0.35** | **-0.83 ± 0.25** |

**Additional file 1:** Parameter estimates (β ± SE) from beta regression models (logit link) evaluating the effects of sample type and STR locus on amplification success (AS), allelic dropout (ADO), and false allele (FA) rates. Blood samples served as the reference sample type in all models. For locus effects, D10S1248 (AS), TPOX (ADO), and CSF1PO (FA) were used as the reference loci because they exhibited the highest values for their respective metrics. Negative coefficients indicate lower values of the response variable relative to the reference, whereas positive coefficients indicate higher values.
