# Supplementary material for: Optimizing a human forensic STR panel for non-invasive genetic monitoring of chimpanzees (Pan troglodytes)
Source: BMC Res Notes. 2026 Sep 9;19:388. doi: 10.1186/s13104-026-07997-y (PMC13721693; doi:10.1186/s13104-026-07997-y)
Supplement: Supplementary file 2 [file 13104_2026_7997_MOESM3_ESM.docx]

| **Locus** | **Amplification success (AS) (emmean ± SE)** | **Allelic dropout (ADO) (emmean ± SE)** | **False allele (FA) (emmean ± SE)** |
| --- | --- | --- | --- |
| D10S1248 | 4.91 ± 0.34 | -4.16 ± 0.42 | -4.74 ± 0.29 |
| CSF1PO | 3.42 ± 0.18 | -2.36 ± 0.23 | -3.08 ± 0.14 |
| D13S317 | 4.58 ± 0.30 | -3.39 ± 0.33 | -4.38 ± 0.25 |
| D16S539 | 3.75 ± 0.21 | -2.71 ± 0.26 | -3.77 ± 0.19 |
| D21S11 | 4.04 ± 0.24 | -3.56 ± 0.34 | -4.15 ± 0.23 |
| D22S1045 | 2.78 ± 0.14 | -3.39 ± 0.35 | -4.74 ± 0.29 |
| D2S1338 | 3.04 ± 0.15 | -3.49 ± 0.33 | -3.36 ± 0.16 |
| D2S441 | 4.58 ± 0.30 | -3.43 ± 0.33 | -4.23 ± 0.24 |
| D3S1358 | 4.43 ± 0.28 | -4.02 ± 0.40 | -4.23 ± 0.24 |
| D5S818 | 3.78 ± 0.21 | -4.20 ± 0.43 | -4.38 ± 0.25 |
| D7S820 | 2.53 ± 0.13 | -2.81 ± 0.25 | -3.71 ± 0.19 |
| D8S1179 | 3.77 ± 0.21 | -3.42 ± 0.32 | -4.00 ± 0.21 |
| FGA | 4.91 ± 0.34 | -3.49 ± 0.33 | -4.15 ± 0.23 |
| TH01 | 4.27 ± 0.26 | -2.18 ± 0.20 | -4.38 ± 0.25 |
| TPOX | 3.25 ± 0.17 | -1.99 ± 0.21 | -3.44 ± 0.16 |
| VWA | 3.99 ± 0.24 | -3.17 ± 0.30 | -3.92 ± 0.20 |

**Additional file 2:** Estimated marginal means (EMMs) for amplification success, allelic dropout (ADO), and false allele (FA) across microsatellite loci derived from generalized linear mixed models (GLMMs). Values are presented as model-predicted marginal means ± standard error (SE) on the logit scale, averaged across sample types. Higher EMM values for amplification success indicate improved amplification performance, whereas lower EMM values for ADO and FA indicate reduced genotyping error rates.
