# Supplementary material for: Optimizing a human forensic STR panel for non-invasive genetic monitoring of chimpanzees (Pan troglodytes)
Source: BMC Res Notes. 2026 Sep 9;19:388. doi: 10.1186/s13104-026-07997-y (PMC13721693; doi:10.1186/s13104-026-07997-y)
Supplement: Supplementary file 3 [file 13104_2026_7997_MOESM4_ESM.docx]

| Individual 1 | Loci typed | Individual 2 | Loci typed | Loci matched | Loci mismatch | Exact/Fuzzy match |
| --- | --- | --- | --- | --- | --- | --- |
| 143R1CMMD | 15 | 149R1CMMD | 15 | 13 | 2 | Fuzzy match |
| 143R1CMMD | 15 | 39R1CMMD | 15 | 13 | 2 | Fuzzy match |
| 143R1CMMD | 15 | 40R1CMMD | 15 | 13 | 2 | Fuzzy match |
| 149R1CMMD | 15 | 39R1CMMD | 15 | 13 | 2 | Fuzzy match |
| 149R1CMMD | 15 | 40R1CMMD | 15 | 13 | 2 | Fuzzy match |
| 30R1CMMD | 16 | 36R1CMMD | 16 | 16 | 0 | Exact match |
| 39R1CMMD | 15 | 40R1CMMD | 15 | 15 | 0 | Exact match |
| CMSY01R1 | 16 | CMSY26R1 | 14 | 13 | 1 | Fuzzy match |

**Additional file 3:** Recapture details of exact and fuzzy match pairs.
